# Trends in Healthcare-Associated Infections and Antimicrobial Use in a Paediatric Hospital from Bucharest, Romania, Across the Pre-Pandemic, Pandemic, and Post-Pandemic Periods (2017–2024)

**DOI:** 10.3390/microorganisms14102278

**Published:** 2026-10-07

**Authors:** Dana Florentina Margine, Carmen-Daniela Chivu, Gabriela-Viorela Nițescu, Bianca Georgiana Enciu, Luisa-Andreea Gheorghe, Andreea Lescaie, Radu Ninel Bălănescu, Alexandru Ulici, Maria Dorina Crăciun, Daniela Pițigoi

**Affiliations:** 1 Department of Epidemiology 1, University of Medicine and Pharmacy “Carol Davila”, 020021 Bucharest, Romania; dana-florentina.margine@drd.umfcd.ro (D.F.M.); luisa-andreea.ilie@drd.umfcd.ro (L.-A.G.); maria.craciun@umfcd.ro (M.D.C.); daniela.pitigoi@umfcd.ro (D.P.); 2 Clinical Emergency Hospital “Sfântul Ioan”, 042122 Bucharest, Romania; 3 Emergency Hospital for Children “Grigore Alexandrescu”, 011743 Bucharest, Romania; viorela.nitescu@umfcd.ro (G.-V.N.); andreea.lescaie@drd.umfcd.ro (A.L.);; 4 Department of Paediatrics, University of Medicine and Pharmacy “Carol Davila”, 020021 Bucharest, Romania; 5 Department of Epidemiology 2, University of Medicine and Pharmacy “Carol Davila”, 020021 Bucharest, Romania; bianca.enciu@umfcd.ro; 6 National Administration of Penitentiaries, 023762 Bucharest, Romania; 7 Department of Paediatric Surgery and Orthopaedics, University of Medicine and Pharmacy “Carol Davila”, 020021 Bucharest, Romania; 8 National Institute of Infectious Diseases “Prof. Dr. Matei Balș”, 021105 Bucharest, Romania

**Keywords:** healthcare-associated infections, antibiotics, point-prevalence survey, paediatric hospitals, COVID-19 pandemic, Romania

## Abstract

The COVID-19 pandemic and the broader epidemiological context may have affected healthcare-associated infections (HAIs) and antimicrobial use (AU), including among the paediatric population. This study used seven point prevalence surveys (PPSs) to characterise HAIs and AU and to observe trends over time at a paediatric hospital in Bucharest. We collected data from 977 patients and identified 31 HAIs in 28 patients with an overall prevalence of 2.9%. During the COVID-19 years, prevalence was higher (4.0%), but no trend was detected. Variables such as surgical ward (aOR: 4.88; CI: 1.63–14.59; *p* = 0.012) and age (aOR: 0.89; CI: 0.81–0.97; *p* = 0.012) were associated with HAI occurrence. AU prevalence was 72.6%, with no specific trend across the study period (*p* = 0.48) overall or by ward. Most prescribed antibiotics were from the Watch category (65.5%), with an upward trend across the study period (*p* < 0.0001), while the Access category registered a downward trend. In conclusion, HAI prevalence did not vary significantly with the epidemiological context. Antibiotic use in the Watch category increased during the pandemic, while the Access category declined. These findings highlight the importance of surveillance for infection prevention and control at the hospital level, and the need for antimicrobial stewardship, guidelines, and continuing medical education on appropriate prescribing practices in paediatric care.

## 1. Introduction

Healthcare-associated infections (HAIs) are an essential indicator of the quality of healthcare services and a major concern in patient safety management. In the European Union/European Economic Area (EU/EEA), HAIs are responsible for 3.5 million cases/year, 90 thousand associated deaths, and 2.5 million disability-adjusted life years (DALYs) [1].

Although Romania reports a lower prevalence of HAIs in the European PPS (3.1% vs. 6.2% in the EU/EEA), it has a markedly higher antimicrobial resistance (AMR) index (68.7% vs. 21.8%). This discrepancy has important implications for healthcare facilities, as the high burden of antimicrobial resistance complicates HAI management and increases the complexity of treatment, infection control, and antimicrobial stewardship interventions [1]. HAIs also increase hospital length of stay (LOS), medical costs, and the risk of severe complications, while approximately one third of the microorganisms associated with HAIs are bacteria resistant to critical antibiotics [2,3].

Paediatric patients are particularly at risk of HAIs due to an immature immune system, the frequent presence of invasive devices, and the length of hospitalisation, which increase the possibility of infection transmission [4,5,6].

These figures may suggest under-reporting and increased antimicrobial use (AU), despite the existing regulations and surveillance programmes [7,8,9,10,11,12].

In this context, additional methods that allow thorough assessment of HAIs and antimicrobial use are necessary for drafting evidence-based infection prevention and control programmes (IPCs). Point prevalence surveys (PPSs) are a simple, rapid, relatively inexpensive tool, that can be repeated over time, allowing the evaluation of HAI prevalence and antimicrobial use at a point in time, identifying areas requiring improvement, and monitoring progress and changes over time. This tool is used worldwide across all types of healthcare settings: acute, chronic, and long-term care facilities (LTCFs) [1,3,13,14]. A recent narrative review on international surveillance practices found that PPS is the most frequently used surveillance method in LTCFs [3].

In 2008, the European Centre for Disease Prevention and Control (ECDC) initiated the Healthcare-Associated Infections Surveillance Network (HAI-Net). Since 2011, the ECDC has conducted PPSs on HAIs and antimicrobial use at five-year intervals across the 27 EU/EEA countries, the United Kingdom, and Serbia, with three survey rounds completed in 2011–2012, 2016–2017, and 2022–2023 [1,14,15,16].

Romania participated in the first ECDC PPS on HAIs and antimicrobial use in 2012. In 2016, following a Ministry of Health Order, annual PPSs were made mandatory for all healthcare facilities with beds, to assess the burden of HAIs and to strengthen the surveillance of antimicrobial use and resistance, thereby supporting the development of effective, evidence-based interventions [3,12,14].

The epidemiological context of the COVID-19 pandemic was a critical factor capable of influencing both the prevalence of HAIs and AU. In Romania, the epidemiological landscape shifted following the first confirmed case of COVID-19 on 26 February 2020. Hospital activities were reorganised and continuously adapted to successive epidemic waves. Infection prevention and control protocols were strengthened in both adult and paediatric healthcare facilities [17,18,19,20,21]. PPSs on HAIs at the hospital level continued to be conducted, except during the first year of the COVID-19 pandemic.

This study aimed to evaluate the trends and characteristics of HAIs and AU across the pre-pandemic (2017–2019), pandemic (2021–2022), and post-pandemic (2023–2024) periods in a paediatric emergency clinical hospital.

## 2. Materials and Methods

### 2.1. Hospital Setting and Participants

The PPSs were conducted at the “Grigore Alexandrescu” Clinical Emergency Hospital for Children, Bucharest, Romania, a tertiary-care paediatric referral centre dedicated exclusively to children from newborns to the age of 18. The hospital is one of the two major paediatric emergency hospitals in the capital, providing continuous multidisciplinary medical and surgical emergency care for children from the capital and across the country. It includes specialised departments for paediatric and plastic reconstructive surgery, as well as a national referral unit for paediatric burn injuries [20,22,23].

During the pandemic, the hospital had a dedicated unit for COVID-19 cases [20]. The hospital participated in the European point prevalence survey of acute care hospitals conducted by ECDC in 2012, 2017 and 2022–2023 [1]. From 2017 to 2025, the hospital’s total capacity varied, and it now has 323 inpatient beds.

Study methodology: descriptive, comparative, univariable, and multivariable analyses were conducted using PPS data collected from 2017 to 2024, in accordance with the methodologies developed by the National Institute of Public Health, which provides the technical coordination of HAI surveillance in Romania. These methodologies were versions of the ECDC original protocols from 2017 and 2022 [24,25,26].

All patients admitted to the facility by 8:00 a.m. on the survey day who had not yet been discharged were eligible for inclusion, except those receiving treatment outside the ward or undergoing surgery on the day of the survey and not yet returned to the ward at the time of data collection. The study excluded outpatients and patients in emergency departments. Newborns in neonatal intensive care or paediatric wards were included if born before 8:00 a.m. on the survey day. The infection prevention and control (IPC) team collected data on a single day in at least one ward. Each year, before the start of the study, the team was instructed on the methodology and the real-world application of the case definitions. We verified active surveillance records from the hospital each year. The total data collection period across all wards did not exceed three weeks each year. We also followed the recommendation to conduct the survey between Tuesday and Friday, due to the typical pattern of Monday admissions.

### 2.2. Data Collection and Management

Data were collected at the hospital, ward, and patient levels using standardised paper forms and subsequently entered in HelicsWin.Net, a free software application provided by the ECDC [24,25].

Data processing included calculating the interval between hospital admission and survey date, as well as the interval between admission and the onset of HAIs. Survey wards were grouped into three categories as follows: non-surgical wards (paediatric wards, cardiology, neurology, toxicology, COVID-19 wards), surgical wards (surgery, neurosurgery, plastic surgery, orthopaedics, and otorhinolaryngology) and intensive care units (ICUs—paediatric intensive care and neonatal intensive care). HAIs and infection sites were classified according to ECDC recommendations [24,25,26]. The PPS data on antimicrobials included systemic antibiotics and antifungal agents. We grouped antibiotics according to the World Health Organisation AWaRe classification (Access, Watch, and Reserve categories) and the Anatomical Therapeutic Chemical (ATC) classification system [27]. The pre-pandemic period and the pandemic periods were defined in relation to the first case of COVID-19 in Romania [17,19,28]. The post-pandemic period was defined as the period following the WHO statement of 5 May 2023, which announced the termination of the COVID-19 Public Health Emergency of International Concern (PHEIC), while recognising that COVID-19 remained an ongoing and established health issue requiring continued long-term management [29,30].

### 2.3. Data Analysis

For the present analysis, patient, antimicrobial use, and HAI data were extracted from the HelicsWin hospital database. Before the analysis, the extracted data were validated both automatically and manually, and two cases were excluded due to incomplete data.

First, we conducted a descriptive analysis to summarise the main characteristics of the data collection process and study population. Afterward, comparative analyses assessed differences in patient characteristics, HAIs and AU prevalence and characteristics across three periods: pre-pandemic (2017–2019), pandemic (2021–2022), and post-pandemic (2023–2024).

We reported HAI prevalence as the percentage of patients with at least one HAI. For HAI types, we reported prevalence using the total number of HAIs as the denominator. We reported AU prevalence as the percentage of patients receiving at least one antimicrobial agent, and we analysed the characteristics of prescriptions across the study period [1,15].

Categorical variables were compared using Pearson’s chi-squared test or Fisher’s exact test, as appropriate. Continuous variables were compared using the Kruskal–Wallis rank sum test. Trends in antimicrobial prescriptions across the predefined study periods were assessed using the Cochran–Armitage test for trend.

For descriptive and comparative analyses, we presented categorical variables as frequencies and percentages, and continuous variables as medians, interquartile ranges (IQRs), means and standard deviations (SDs).

We conducted univariable and multivariable logistic regression analyses and reported odds ratios (ORs), adjusted odds ratios (aORs), 95% confidence intervals (95% CIs), and *p*-values to identify factors associated with HAIs and antimicrobial use. No epidemiological clusters or outbreaks were detected within the wards. Variables with a *p*-value less than 0.1 in the univariable analysis or other relevant variables were included in the multivariable model [31,32,33]. Multicollinearity among the independent variables was assessed using the variance inflation factor (VIF). Model fit was evaluated by comparing the multivariable model with the null model using the likelihood-ratio chi-square test, together with the Akaike information criterion (AIC) and Bayesian information criterion (BIC). Model discrimination was assessed using the area under curve (AUC). The model explanatory power was summarised using Nagelkerke’s adjustments to Cox and Snell’s pseudo-*R*^2^ values. Variables with *p*-values less than 0.05 were considered statistically significant. Microsoft Excel for 365, R Studio/2026.07.0 Build 147 software, and JASP 0.98.0 version (JASP Team, 2026, Amsterdam, The Netherlands) were used for data processing, analyses, and visualisation.

### 2.4. Ethical Considerations

This study was conducted in accordance with the principles of the Declaration of Helsinki and the applicable national regulations. The Ethics Committee of the Clinical Emergency Hospital for Children “Grigore Alexandrescu” approved the use of PPS data for this retrospective study (approval number: 26288/12.08.2025). Due to the study’s retrospective nature, only standard informed consent was required. It was obtained from the participants’ parents and legal guardians at admission and included consent for the use of clinical and surveillance data for academic purposes, given the facility’s additional status as a teaching hospital.

Patient information was anonymised during data collection, uploading, and analysis using anonymous identifiers. Access to data was limited to the IPC team and study investigators, ensuring confidentiality.

## 3. Results

PPS data were collected over a 7-year period: in 2017 (6 July–10 August), 2018 (2–22 August), 2019 (22 July–13 August), 2021 (27 July–11 August), 2022 (16–24 August), 2023 (30 May–14 June) and 2024 (30 July–14 August).

It included a total of 977 patients grouped into three periods (pre-pandemic—422 (43.2%) patients; pandemic—253 (25.9%) patients; and post-pandemic: 302 (30.9%) patients). The 16 wards were grouped into three categories: non-surgical (8 wards, 396 patients; 40.5%), surgical (6 wards, 502 patients; 51.4%) and ICU (2 wards, 79 patients; 8.1%). Patient distribution across the study period showed no statistically significant variation by ward and reflected the hospital surgical profile.

The demographic characteristics showed a median age of 4.5 years (IQR: 1.0–11.0; mean: 6.1; SD: 5.7), with a statistically significant younger age during the pandemic period (median: 3.0 years; IQR: 0.0–10.0; mean: 5.2; SD: 5.5; *p* = 0.002). For children younger than 2 years (*n* = 324), age was expressed in months, with a median of 5.0 months (IQR: 1.0–11.0; mean: 7.0; SD: 6.0), and no statistically significant variations were registered across periods (*p* = 0.2). A slight predominance of males was observed (577; 59.1%), with no statistically significant differences between periods (*p* = 0.2).

The median number of days between admission and survey was 3.0 days (IQR: 1.0–8.0; mean: 11.0; SD: 35.0), with no statistical difference between periods (Table 1).

### 3.1. HAI Prevalence and Characteristics

Twenty-eight (2.9%) patients presented at least one HAI (7 in the pre-pandemic period, 10 in the pandemic period and 11 in the post-pandemic period). Three patients presented two concomitant HAIs, resulting in a total of 31 HAIs identified during the study period. Two patients with two concomitant HAIs were found during the pandemic, and one in the post-pandemic period.

HAI prevalence increased from 1.7% (95% CI: 0.7–3.4) in the pre-pandemic period to 4.0% (95% CI: 1.9–7.3) in the pandemic, followed by a slight decrease to 3.6% (95% CI: 1.8–6.5) in the post-pandemic period; however, no statistically significant temporal trend was observed, as presented in Table 2.

HAI prevalence by ward varied over the period but did not reach the statistical significance level. The highest prevalence values were registered in ICU and surgical wards.

**Figure 1:**
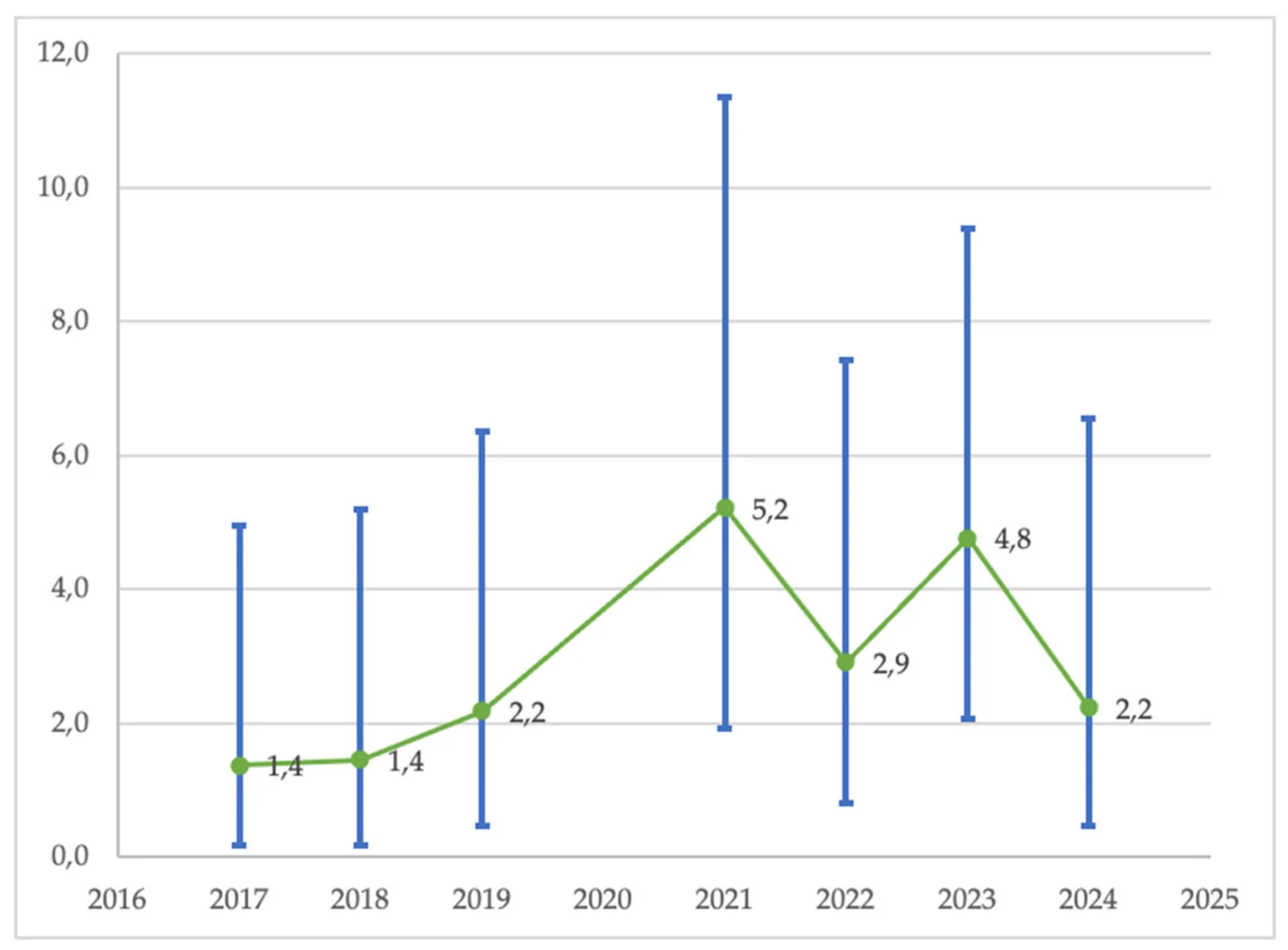
Annual prevalence of HAIs with 95% confidence intervals, 2017–2024.

Figure 1 describes year-to-year variation in annual HAI prevalence, with wide and overlapping confidence intervals.

Surgical site infections were the most prevalent HAI diagnosis (0.9%), followed by bloodstream infections (0.8%). In the pre-pandemic period, the most prevalent HAI was skin and soft tissue infections (0.5%), while during the pandemic, bloodstream infections were more prevalent (2.0%). In the post-pandemic period, the most prevalent HAI was surgical site infection (1.3%), followed by bloodstream infections (0.7%), gastro-intestinal infections (0.7%), and respiratory tract infections (0.7%). Detailed data on HAI types are presented in Table 3.

Of the 11 cases of pneumonia and bloodstream infections (including clinical sepsis in neonates) monitored for device association, 7 (63.6%) had a relevant device.

Almost all HAIs were associated with the current hospitalisation, except for one recorded during the pandemic period, which was associated with previous hospitalisation in LTCF. The shortest time interval between HAI onset and survey date was recorded for the pandemic period (median: 5.0; IQR: 3.0, 8.0; mean: 6.8; SD: 5.5) but it was not a statistically supported difference (*p* = 0.8).

During the pre-pandemic period, *Pseudomonas aeruginosa* was the most frequently isolated pathogen (3/7 isolates, 42.9%), followed by *Staphylococcus aureus* (2/7, 28.6%). During the pandemic period, *Klebsiella* spp. predominated (5/11, 45.5%), while in the post-pandemic period, the microbiological spectrum became more heterogeneous, with several pathogens being isolated only once (Table 4). Four cases of HAIs had no identified etiological agent.

Univariable analysis identified age (years) (OR 0.91; 0.83–0.98) and surgical wards (OR 4.28; 1.61–14.80) as statistically significant factors. The logistic regression also identified age and surgical ward as statistically significant factors in the model (Table 5).

The full model containing all independent variables had pseudo-R^2^ values of 0.026 (Cox and Snell R^2^) and 0.114 (Nagelkerke R^2^). The multivariable logistic regression model was significantly better than the null model (likelihood-ratio χ^2^ (4) = 25.949, *p* < 0.001). The AIC was lower for the multivariable model than for the null model (238.168 vs. 256.118), whereas the BIC was slightly higher (262.591 vs. 261.002).

Model discrimination was acceptable, with an AUC of 0.77. No multicollinearity was detected, with all variance inflation factor (VIF) values between 1.1 and 1.2.

Having been admitted to a surgical ward was associated with higher odds of HAI occurrence (aOR: 4.88; 95% CI: 1.63–14.59), though the confidence interval was relatively broad. In contrast, age was statistically significantly associated with lower odds of HAI occurrence both in univariable and multivariable analyses, with an OR and aOR of 0.91 (0.83–0.98) and 0.89 (0.82–0.97). These data suggest the need to identify other factors in these subgroups and to further complete the research.

### 3.2. Antimicrobial Use Characteristics

Of the total number of patients included in the study, 709 (72.6%) received at least one antimicrobial at the time of the survey, with the highest proportion during the pandemic period (191; 75.5%), but this difference was not statistically significant (*p* = 0.48). The data are presented in Table 6.

**Figure 2:**
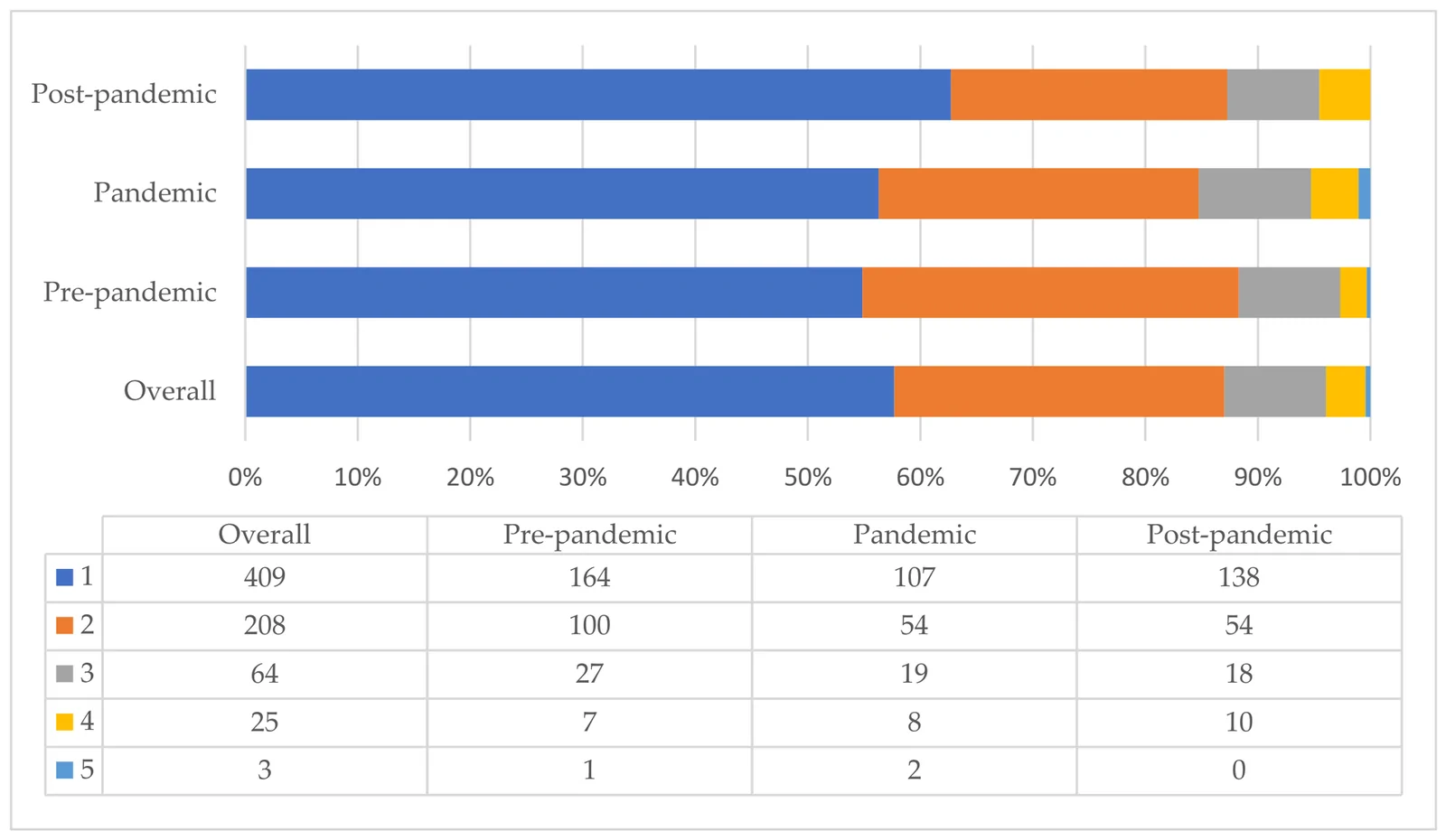
Distribution of the number of antibiotic classes received, across the study period (2017–2024).

According to ATC classification, 409 patients (41.9%) received one antimicrobial, 208 (21.3%) received prescriptions with two classes, 64 (6.6%) received three classes, 25 (2.6%) received four classes, and 3 (0.3%) received five classes, while 268 patients (27.4%) received no antimicrobials. The data are presented graphically in Figure 2.

Antibiotics were the most prescribed antimicrobials (1081; 95.2%). Most antibiotics used were in the World Health Organisation’s Watch category (708; 65.5%), with an upward trend across the study period (*p* < 0.0001). The Access category registered a downward trend across the pandemic and post-pandemic period (*p* < 0.0001). The Reserve and Not Recommended categories had variations without statistical significance.

Most antimicrobials were administered via the parenteral route (1077; 94.9%), with no statistically significant difference between periods (*p* = 0.40). Most antimicrobials were used for community-acquired infection treatment (522; 46.0%), followed by surgical prophylaxis for more than 1 day (320; 28.2%), medical prophylaxis (144; 12.7%) and HAI treatment (51; 4.5%). Medical prophylaxis, one-day surgical prophylaxis, and >1-day surgical prophylaxis registered statistically significant variations across the study period, with significant descending trends for surgery.

The reason for antimicrobial use was mentioned for 1090 (96.0%) antimicrobials, with a slightly lower percentage in the pandemic period (293; 93.3%; *p* < 0.0005).

The highest proportion of antimicrobial prescriptions was in surgical wards (632; 55.7%) with statistically significant variations across the study period (*p* = 0.004). During the COVID-19 pandemic, a statistically significant higher rate of antimicrobial prescriptions was identified in ICUs (56; 17.8%, *p* = 0.0001), as presented in Table 7.

Pneumonia was the most frequent diagnosis in antimicrobial prescriptions (161; 14.2%), followed by otolaryngotracheal infections (90; 7.9%) and intra-abdominal sepsis (70; 6.2%). Pneumonia (41; 13.1%), intra-abdominal sepsis (27; 8.6%), and gastro-intestinal infections (25; 8.0%) were mostly linked to antimicrobial prescribing during the pandemic (Table 8).

In the univariable analysis, the ICU ward variable was statistically significant.

Logistic regression identified the age variable as having marginal statistical significance, and with confidence intervals containing 1. Only ICU presented an aOR of 2.23 (95%CI: 1.16–4.28; *p* = 0.016) (Table 9). The full model containing all independent variables had pseudo-R^2^ values of 0.012 (Cox and Snell R^2^) and 0.018 (Nagelkerke R^2^). The multivariable logistic regression model was significantly better than the null model (likelihood-ratio χ^2^ (2) = 12.280, *p* = 0.015). The AIC was lower for the multivariable model than for the null model (1145.691 vs. 1149.971), whereas the BIC was higher (1170.113 vs. 1154.855).

Model discrimination was low, with an AUC of 0.55. No multicollinearity was detected, with all variance inflation factor (VIF) values between 1.1 and 1.2.

## 4. Discussion

In this study, we evaluated the trends in HAIs and antimicrobial use in one of the largest paediatric hospitals in Romania over a 7-year period. Using PPS data, we compared three distinct epidemiological periods (pre-pandemic, pandemic, and post-pandemic), finding that the prevalence of HAIs remained relatively stable over time despite the challenges imposed by the COVID-19 pandemic. The studies were conducted during the summer months to avoid seasonal variation in HAIs, although differences in survey timing within the summer period cannot be excluded. In 2020, the hospital was structurally reorganised as a national preparedness plan for the COVID-19 pandemic and no PPS was conducted [21]. During the study period, demographics showed statistically significant differences only for age, with younger patients being admitted during the pandemic. The HAI prevalence was 2.9%, similar to the HAI prevalence identified in the 2022 national PPS, with no significant trend across periods [1]. Other PPSs, undertaken in Greek paediatric hospitals in 2022, reported a prevalence of 3.5%, which is comparable to our findings [34]. HAI prevalence varied by ward across the study, but no significant trend was identified.

Most HAIs were identified in surgical wards and were bloodstream infections and surgical site infections. This pattern reflects the hospital’s profile, with multiple specialised surgical departments providing advanced paediatric surgical care [20,22,23]. The aetiology was most often bacterial, with *Klebsiella pneumoniae* and *Pseudomonas aeruginosa* the most frequent isolates. The hospital admitted COVID-19 patients during the pandemic and post-pandemic period, but only one COVID-19 case was registered as an HAI. All PPSs were conducted in months with a low incidence of COVID-19 in the community [17,18,19,35].

Regarding factors associated with HAI occurrence, increased age was associated with lower odds of HAI while surgical wards were associated with higher odds of HAI occurrence. These findings suggest the need for increased awareness and strengthened HAI prevention measures in neonates and infants, who represent the most vulnerable patient population.

AU prevalence was high (72.6%) but stable across the periods, with no statistically significant trends overall or by ward as most patients enrolled in PPSs had at least one ATC class prescribed.

Excessive antimicrobial use also increases the risk of infections caused by *Clostridioides difficile*. For example, a Romanian study published in 2024 highlighted that 61.3% of *Clostridioides difficile* infections hospitalised over a 4-year period in a tertiary infectious disease hospital in Bucharest used antibiotics in the three months before hospitalisation [36]. In our study, there was only one case of *Clostridioides difficile* infection, registered as HAI in the post-pandemic period, but the prolonged impact of antibiotic use could not be assessed in the emergency hospital.

The AU prevalence was higher than 40.9%, as reported in the United Kingdom, in a study conducted across 61 paediatric units, and a PPS study conducted in Greece in 2022, which found a 39.5% AU prevalence in paediatric hospitals [34,37].

A US study found that about one in three hospitalised children will get at least one antibiotic during their hospital stay [38]. These comparisons should be interpreted with caution due to differences in the epidemiological context of bacterial infections and antimicrobial resistance between countries, as well as variations in study periods, and the profile of units included in the study. Romania registered an increasing number of cases of whooping cough and measles [39,40]. However, antibiotic use varies widely by hospital type and clinical setting, with the highest rates usually seen in paediatric intensive care units, neonatal intensive care units, and surgical wards [41,42]. This observation is consistent with both the published literature and our study findings.

AU in Romania remains higher than in many other OECD countries, and, unfortunately, our country is currently not on track to achieve its 2030 antimicrobial consumption reduction goal [43]. The use of these antibiotics has led to an increase in bacterial resistance, with the antimicrobial resistance in Romania being approximately three times the EU/EEA average [1].

The study found a lower proportion of antimicrobials prescribed for medical prophylaxis (12.7% vs. 17.3%) and a higher proportion prescribed for surgical prophylaxis (33.9% vs. 6.8%) than the abovementioned study conducted in the United Kingdom. These findings further support the hypothesis that the differences observed could also be attributable to the distinct clinical profiles of the included paediatric units [37].

The hypothesis that the observed differences could be attributable to the study periods is supported by a study that compared PPS data regarding antibiotic use in the hospital in 2012 and 2022, which showed a slight increase in antibiotic use in 2022 (73.9%) compared with 2012 (71.0%) [44].

Although the hospital transformed from 2017 to 2024 to provide more complex medical care, including hepatic transplantation, its profile remained that of an acute centre for paediatric specialised care.

It is worth mentioning that, despite the high use of antimicrobials for various types of prophylaxis or HAI treatment, almost half of the antimicrobials were used for community-acquired infections, according to the hospital profile, and similar to a PPS conducted in 2023, in a paediatric tertiary hospital from the Republic of Panama [45].

The types of antibiotics differed by AWaRE categories from other studies and WHO recommendation regarding using at least 60% from the Access category. In our study, the trends of AU were downward for antibiotics in the Access category (AU prevalence 26.5%) and upward for the Watch category (AU prevalence 65.5%), similar to a PPS conducted in Greece, 2022. Another study, mentioned above, reported AU prevalence for the Access category of 68.2% [27,34,45]. These findings are strongly supported by the high level of antibiotic resistance observed in Romania and the hospital’s profile, which includes tertiary, emergency, and specialised patients [20,22,23,46].

Beyond quantifying AU, a comprehensive evaluation of prescribing practices should also assess the appropriateness of antibiotic use. This includes comparing prescriptions with evidence-based clinical guidelines and determining whether the prescribed antimicrobial therapy is consistent with the documented clinical indications. Such assessments are essential for identifying opportunities to optimise antibiotic prescribing and strengthen antimicrobial stewardship interventions [47].

*Strengths:* to our knowledge, it is one of the first studies in Romania to provide data on HAIs and AU PPSs, over a 7-year period, and to focus on the paediatric population. Because paediatric hospitals accounted for only 6% of European PPSs conducted in 2016–2017 and 2022–2023, the results of this study complement the general European PPS findings by providing insights and details on HAIs and AU from an emergency surgical hospital.

All the PPSs included in the analysis were conducted by ECDC-standardised protocols, which minimises any selection biases [25,26].

The findings reveal both notable similarities and distinct differences when compared to the existing literature, underscoring the dynamics and value of active HAI surveillance data and highlighting that epidemiological data must be interpreted within the clinical and structural context of healthcare facilities.

*Limitations:* the study comprises multiple cross-sectional studies, which are not designed to capture HAI epidemiological processes and clusters. To address this issue, the team verified the HAI active surveillance records. The PPSs were conducted in one Romanian paediatric hospital; thus, results may not apply elsewhere without identifying specific differences between hospitals. However, the study provides sufficient data on patient characteristics and demographics and describes the hospital thoroughly for specific comparison. Data from 2020 were unavailable because PPSs could not be conducted. However, these data are not comparable with other pandemic years, as admissions were limited, and the wards were reorganised under the COVID-19 pandemic preparedness plan. During the study period, we observed a small number of HAIs. However, this cannot be stated as under-detection, based on demographics in the emergency hospitals (median LOS of 3 days). In the logistic regression, we limited the predictors to three parameters, resulting in an event-per-variable ratio of 9.3. The confidence intervals were wide, limiting the precision of the estimates.

Although all surveys were conducted during the summer months, differences in survey timing may have introduced some differences. Changes in hospital activity and clinical complexity over time may have also influenced the observed results. Patient-level antimicrobial resistance data were not available, and the appropriateness of prescriptions was not assessed. Finally, the cross-sectional design of the study limits the ability to establish temporality between potential associated factors and HAI occurrence, and the findings may have limited generalisability beyond the study hospital.

Despite all these limitations, PPSs are an efficient tool for surveying HAI and AU factors that necessitate complementary interventions. The study’s results have proven useful for explaining the context of the topic and for supporting recommendations to improve surveillance of HAIs, AU, and antimicrobial resistance, including continuing the annual PPS and expanding it to LTCFs. This is relevant since Romania has not participated in any of the PPSs conducted across European LTCFs [3].

Surveillance data can guide interventions and public health policies towards IPCs, antimicrobial stewardship programmes and a continuous medical education based on paediatric guidelines kept up to date with the epidemiological context.

## 5. Conclusions

In conclusion, the current study underscores the utility of repeated PPSs in monitoring healthcare-associated infection trends and antimicrobial use in a large emergency clinical paediatric hospital in Romania over a 7-year period. HAIs and AU prevalence did not vary significantly across periods. The antimicrobial prescribing practices registered a downward trend for the Access category and an upward trend for the Watch category. These findings highlight the importance of surveillance for infection prevention and control programmes, and the need for antimicrobial stewardship, guidelines, and continuing medical education on appropriate prescribing practices in paediatric care.

## Figures and Tables

**Table 1:** Descriptive and comparative analysis of PPS patient characteristics, 2017–2024.

Characteristic	Overall	Pre-Pandemic	Pandemic	Post-Pandemic	*p*-Value ^4^
	*N* = 977 ^1^	*n* = 422 ^1^ (43.2)	*n* = 253 ^1^ (25.9)	*n* = 302 ^1^ (30.9)	
**Ward specialty**					0.2
Non-surgical	396 (40.5%)	171 (40.5%)	108 (42.7%)	117 (38.7%)	0.64
Surgical	502 (51.4%)	221 (52.4%)	117 (46.2%)	164 (54.3%)	0.13
ICU	79 (8.1%)	30 (7.1%)	28 (11.1%)	21 (7.0%)	0.14
**Age (years)**					0.002
Mean (SD)	6.1 (5.7)	6.6 (5.7)	5.2 (5.5)	6.3 (5.8)	
Median (IQR)	4.5 (1.0–11.0)	6.0 (1.0–12.0)	3.0 (0.0–10.0)	5.0 (1.0–12.0)	
**Age (months) ^2^**					0.2
Mean (SD)	7.0 (6.0)	8.0 (7.0)	6.0 (6.0)	6.0 (6.0)	
Median (IQR)	5.0 (1.0–11.0)	7.0 (1.0–13.0)	4.0 (1.0–10.0)	5.0 (2.0–9.0)	
**Gender**					0.2
Female	400 (40.9%)	187 (44.3%)	95 (37.5%)	118 (39.1%)	
Male	577 (59.1%)	235 (55.7%)	158 (62.5%)	184 (60.9%)	
**Length of hospital stay (LOS) ^3^**					0.2
Mean (SD)	11.0 (35.0)	10.0 (41.0)	14.0 (36.0)	10.0 (25.0)	
Median (IQR)	3.0 (1.0–8.0)	3.0 (1.0–8.0)	3.0 (1.0–8.0)	2.0 (1.0–7.0)	

^1^ *N*—number of patients in the entire study; *n* (%)—number of patients in subgroups; ^2^ ages under 2 years; ^3^ LOS before the PPS; IQR—interquartile range; SD—standard deviation; ^4^ Pearson’s chi-squared test; Kruskal–Wallis rank sum test.

**Table 2:** Descriptive and comparative analysis of HAI prevalence, 2017–2024.

Characteristic	Overall	Pre-Pandemic	Pandemic	Post-Pandemic	*p*-Value ^3^
	*N* = 977 ^1^	*n* = 422 ^1^ (43.2)	*n* = 253 ^1^ (25.9)	*n* = 302 ^1^ (30.9)	
**HAI Prevalence ^2^**	2.9 (2.0–4.1)	1.7 (0.7–3.4)	4.0 (1.9–7.3)	3.6 (1.8–6.5)	0.14
**HAI Prevalence by Ward ^2^**					
**Non-Surgical**	1.0 (0.6–3.9)	0.6 (0.2–4.7)	1.9 (1.0–12.8)	0.9 (0.2–5.9)	0.57
**Surgical**	4.2 (3.2–7.0)	2.3 (1.0–5.2)	7.7 (4.1–14)	6.1 (3.4–10.9)	0.12
**ICU**	3.8 (1.3–10.6)	3.3 (0.6–16.7)	3.6 (0.6–17.7)	4.8 (0.9–22.7)	1.00

^1^ *N*—number of patients in the entire study; *n* (%)—number of patients in subgroups; ^2^ 95% confidence interval; ^3^ Pearson’s chi-squared test.

**Table 3:** Prevalence of healthcare-associated infections by infection type across the pre-pandemic, pandemic, and post-pandemic periods.

Characteristic	Overall	Pre-Pandemic	Pandemic	Post-Pandemic
	*n* = 31 ^1^	*n* = 7 ^1^	*n* = 12 ^1^	*n* = 12 ^1^
**Pneumonia and respiratory tract infections**	**3 (0.3%)**	**0 (0.0%)**	**1 (0.4%)**	**2 (0.7%)**
Pneumonia	2 (0.2%)	0 (0.0%)	1 (0.4%)	1 (0.3%)
COVID-19	1 (0.1%)	0 (0.0%)	0 (0.0%)	1 (0.3%)
**Surgical site infection**	**9 (0.9%)**	**1 (0.2%)**	**4 (1.6%)**	**4 (1.3%)**
Superficial	3 (0.3%)	1 (0.2%)	1 (0.4%)	1 (0.3%)
Deep	1 (0.1%)	0 (0.0%)	1 (0.4%)	0 (0.0%)
Organ	4 (0.4%)	0 (0.0%)	1 (0.4%)	3 (1.0%)
Unspecified	1 (0.1%)	0 (0.0%)	1 (0.4%)	0 (0.0%)
**Bloodstream infection**	**8 (0.8%)**	**1 (0.4%)**	**5 (2.0%)**	**2 (0.7%)**
**Clinical sepsis in neonates**	**1 (0.1%)**	**0 (0.0%)**	**0 (0.0%)**	**1 (0.3%)**
**Gastro-intestinal system infection**	**4 (0.4%)**	**1 (0.2%)**	**1 (0.4%)**	**2 (0.7%)**
*Clostridioides difficile* infection	1 (0.1%)	0 (0.0%)	0 (0.0%)	1 (0.3%)
Other gastro-intestinal system infection	3 (0.3%)	1 (0.2%)	1 (0.4%)	1 (0.3%)
**Skin and soft tissue infection**	**2 (0.2%)**	**2 (0.5%)**	**0 (0.0%)**	**0 (0.0%)**
**Burn Infection**	**2 (0.2%)**	**1 (0.2%)**	**0 (0.0%)**	**1 (0.3%)**
**Central nervous system infection**	**1 (0.1%)**	**0 (0.0%)**	**1 (0.4%)**	**0 (0.0%)**
**Mouth infection**	**1 (0.1%)**	**1 (0.2%)**	**0 (0.0%)**	**0 (0.0%)**

^1^ *n*—number of HAIs.

**Table 4:** Distribution of microbiological isolates associated with healthcare-associated infections across the pre-pandemic, pandemic, and post-pandemic study periods.

Microorganism	Total Isolates	Pre-Pandemic	Pandemic	Post-Pandemic
**Bacteria**				
*Klebsiella* spp.	7	1	5	1
*Pseudomonas aeruginosa*	5	3	1	1
*Acinetobacter* spp.	1	0	0	1
*Enterococcus faecium*	1	0	0	1
*Staphylococcus aureus*	4	2	2	0
*Coagulase-negative staphylococci* (other CNS)	1	0	0	1
*Streptococcus* spp. (other)	2	0	1	1
*Stenotrophomonas maltophilia*	1	0	0	1
*Clostridioides difficile*	1	0	0	1
**Fungi**				
*Candida* spp.	1	0	1	0
**Viruses**				
Norovirus	1	1	0	0
Respiratory syncytial virus	1	0	1	0
SARS-CoV-2	1	0	0	1
**Total isolates**	27	7	11	9

**Table 5:** Factors associated with occurrence of HAIs, assessed in univariable and multivariable analyses, 2017–2024.

Characteristic	Univariable Analysis		Multivariable Analysis	
	OR (95% CI)	*p*-Value	aOR (95% CI)	*p*-Value
**Age (years)**	0.91 (0.83–0.98)	0.020	0.89 (0.82–0.97)	0.012
**Ward specialty**				
Non-Surgical	Reference		Reference	
Surgical	4.28 (1.61–14.80)	0.008	4.88 (1.63–14.59)	0.005
ICU	3.87 (0.75–17.90)	0.080	1.69 (0.30–9.46)	0.549
**Hospitalisation days**	1.01 (1.00–1.01)	0.004	1.01 (1.00–1.01)	0.008

CI—confidence interval, OR—odds ratio, aOR—adjusted odds ratio.

**Table 6:** Descriptive and comparative analysis of overall antimicrobial use and by ward, in the study period (2017–2024).

Characteristic	Overall	Pre-Pandemic	Pandemic	Post-Pandemic	*p*-Value ^3^
	*N* = 977 ^1^	*n* = 422 ^2^ (43.2)	*n* = 253 ^2^ (25.9)	*n* = 302 ^2^ (30.9)	
**AU Prevalence**	72.6 (69.7–75.4)	70.9 (66.3–75.2)	75.1 (69.3–80.3)	72.8 (67.5–77.8)	0.48
**AU prevalence by Ward ^2^**					
**Non-Surgical**	69.9 (61.9–78.7)	64.9 (53.4–78.7)	71.3 (56.27–89.1)	76.1 (61.1–93.6)	0.12
**Surgical**	73.3 (66.0–81.2)	75.1 (64.1–87.5)	74.4 (59.6–91.7)	70.1 (57.9–84.2)	0.97
**ICU**	82.3 (63.5–100)	76.7 (48.6–100)	92.9 (60.0–100)	76.2 (43.6–100)	0.19

^1^ *N*—number of patients in the entire study; *n* (%)—number of patients in subgroups; ^2^ 95% confidence interval; ^3^ Pearson’s chi-squared test.

**Table 7:** Descriptive and comparative analyses of the antimicrobial utilisation pattern, 2017–2024.

Characteristic	Overall	Pre-Pandemic	Pandemic	Post-Pandemic	*p*-Value ^2^
	*N* = 1135 ^1^	*n* = 481 ^1^	*n* = 314 ^1^	*n* = 340 ^1^	
**Antimicrobials**					0.3
Antibiotics	1081 (95.2%)	462 (96.0%)	294 (93.6%)	325 (95.6%)	
Antifungal	54 (4.8%)	19 (4.0%)	20 (6.4%)	15 (4.4%)	
**AWARE**					
Access	286 (26.5%)	145 (31.4%)	71 (24.1%)	70 (21.6%)	<0.0001 (<0.0001) ^3^
Watch	708 (65.5%)	289 (62.5%)	198 (67.4%)	221 (68.0%)	<0.0001 (<0.0001) ^3^
Reserve	79 (7.3%)	28 (6.1%)	23 (7.8%)	28 (8.6%)	0.71
Not recommended	8 (0.7%)	0 (0.0%)	2 (0.7%)	6 (1.8%)	–
**Route of administration**					0.2
Inhalation	2 (0.2%)	0 (0.0%)	2 (0.6%)	0 (0.0%)	–
Oral	56 (4.9%)	26 (5.4%)	17 (5.4%)	13 (3.8%)	0.49
Parenteral	1077 (94.9%)	455 (94.6%)	295 (93.9%)	327 (96.2%)	0.40
**Indication for Antimicrobial Use**					
Treatment of community-acquired infections	522 (46.0%)	205 (42.6%)	160 (51.0%)	157 (46.2%)	0.07
Treatment of LTCF-associated infections	2 (0.2%)	0 (0.0%)	2 (0.6%)	0 (0.0%)	–
HAI treatment	51 (4.5%)	15 (3.1%)	18 (5.7%)	18 (5.3%)	0.15
Medical prophylaxis	144 (12.7%)	59 (12.3%)	27 (8.6%)	58 (17.1%)	0.005
Single-dose surgical prophylaxis	18 (1.6%)	6 (1.2%)	8 (2.5%)	4 (1.2%)	0.27
One-day surgical prophylaxis	47 (4.1%)	24 (5.0%)	17 (5.4%)	6 (1.8%)	0.002 (0.03) ^3^
>1-day surgical prophylaxis	320 (28.2%)	169 (35.1%)	59 (18.8%)	92 (27.1%)	<0.0001 (0.004) ^3^
Other indication	21 (1.9%)	1 (0.2%)	19 (6.1%)	1 (0.3%)	<0.0001
Unknown/missing	10 (0.9%)	2 (0.4%)	4 (1.3%)	4 (1.2%)	0.35
**Reason mentioned**					
No	45 (4.0%)	7 (1.5%)	21 (6.7%)	17 (5.0%)	
Yes	1090 (96.0%)	474 (98.5%)	293 (93.3%)	323 (95.0%)	<0.0005
**Ward specialty**					<0.001
Non-Surgical	376 (33.1%)	154 (32.0%)	107 (34.1%)	115 (33.8%)	0.79
Surgical	632 (55.7%)	288 (59.9%)	151 (48.1%)	193 (56.8%)	0.004
ICU	127 (11.2%)	39 (8.1%)	56 (17.8%)	32 (9.4%)	0.0001

^1^ *N*—number of antimicrobial prescriptions in the entire study; *n* (%)—number of prescriptions in subgroups; ^2^ Pearson’s chi-squared test; Fisher’s exact test; () ^3^ *p*-value for Cochran–Armitage test for trends.

**Table 8:** Indication for antimicrobial use as treatment, by the site of diagnosis, 2017–2024.

Characteristic	Overall	Pre-Pandemic	Pandemic	Post-Pandemic
	*N* = 1132 ^1^	*n* = 478 ^1^	*n* = 314 ^1^	*n* = 340 ^1^
**Respiratory tract**				
Pneumonia	161 (14.2%)	66 (13.7%)	41 (13.1%)	54 (15.9%)
Acute Bronchitis	18 (1.6%)	2 (0.4%)	11 (3.5%)	5 (1.5%)
Cystic fibrosis	6 (0.5%)	5 (1.0%)	1 (0.3%)	0 (0.0%)
**Urinary tract**				
Symptomatic upper UTI	19 (1.7%)	10 (2.1%)	5 (1.6%)	4 (1.2%)
Symptomatic lower UTI	19 (1.7%)	10 (2.1%)	2 (0.6%)	7 (2.1%)
**Systemic infections**				
Lab-confirmed bacteriaemia	33 (2.9%)	2 (0.4%)	16 (5.1%)	15 (4.4%)
Clinical sepsis	12 (1.1%)	6 (1.2%)	6 (1.9%)	0 (0.0%)
Systematic inflammatory response with no clear anatomic site	3 (0.3%)	2 (0.4%)	0 (0.0%)	1 (0.3%)
**Intra-abdominal sepsis**	70 (6.2%)	29 (6.0%)	27 (8.6%)	14 (4.1%)
**Gastro-intestinal infection**	59 (5.2%)	12 (2.5%)	25 (8.0%)	22 (6.5%)
**Surgical site infection**	6 (0.5%)	2 (0.4%)	4 (1.3%)	0 (0.0%)
Cellulitis wound, deep soft tissue not involving the bone	40 (3.5%)	12 (2.5%)	12 (3.8%)	16 (4.7%)
Otolaryngotracheal infections	90 (7.9%)	50 (10.4%)	15 (4.8%)	25 (7.4%)
**Central nervous system**	12 (1.1%)	1 (0.2%)	4 (1.3%)	7 (2.1%)
**Cardiovascular system**	4 (0.4%)	0 (0.0%)	0 (0.0%)	4 (1.2%)
**Other**				
Septic arthritis, not related to surgery	8 (0.7%)	8 (1.7%)	0 (0.0%)	0 (0.0%)
Epididymo-orchitis	3 (0.3%)	3 (0.6%)	0 (0.0%)	0 (0.0%)
Gynaecological infections	4 (0.4%)	0 (0.0%)	3 (1.0%)	1 (0.3%)
Unknown	7 (0.6%)	0 (0.0%)	6 (1.9%)	1 (0.3%)
Undefined, site with no systemic inflammation	2 (0.2%)	0 (0.0%)	2 (0.6%)	0 (0.0%)

^1^ *N*—number of antimicrobial prescriptions in the entire study; *n* (%)—number of prescriptions in subgroups.

**Table 9:** Factors associated with antimicrobial use, assessed in univariable and multivariable analyses, 2017–2024.

Characteristic	Univariable Analysis		Multivariable Analysis	
	OR (95% CI)	*p*-Value	aOR (95% CI)	*p*-Value
**Age (years)**	0.98 (0.95–1.00)	0.057	0.97 (0.95–1.00)	0.045
**Ward specialty**				
Non-Surgical	Reference		Reference	
Surgical	1.22 (0.91–1.63)	0.2	1.31 (0.97–1.77)	0.075
ICU	2.04 (1.13–3.92)	0.02	2.23 (1.16–4.28)	0.016
**Hospitalisation days**	1.00 (1.00–1.01)	0.5	1.00 (0.99–1.00)	0.101

CI—confidence interval, OR—odds ratio, aOR—adjusted odds ratio.

## Data Availability

The datasets generated and analysed during the current study are available from the corresponding author upon reasonable request.
